# Drug repurposing as an alternative for the treatment of recalcitrant bacterial infections

**DOI:** 10.3389/fmicb.2015.00282

**Published:** 2015-04-09

**Authors:** Adrián Rangel-Vega, Lawrence R. Bernstein, Edna Ayerim Mandujano-Tinoco, Silvia Julieta García-Contreras, Rodolfo García-Contreras

**Affiliations:** ^1^Department of Microbiology and Parasitology, Faculty of Medicine, Universidad Nacional Autónoma de MéxicoMexico City, Mexico; ^2^Terrametrix, CAUSA; ^3^Epigenetics of Cancer Laboratory, Division of Basic Research, National Institute of Genomic MedicineMexico City, Mexico; ^4^Centro Médico Coyoacán, Mexico CityMexico

**Keywords:** gallium, 5-fluorouracil, ciclopirox, diflunisal, bacterial infections, drug repurposing

## Abstract

Bacterial infection remains one of the leading causes of death worldwide, and the options for treating such infections are decreasing, due the rise of antibiotic-resistant bacteria. The pharmaceutical industry has produced few new types of antibiotics in more than a decade. Researchers are taking several approaches toward developing new classes of antibiotics, including (1) focusing on new targets and processes, such as bacterial cell–cell communication that upregulates virulence; (2) designing inhibitors of bacterial resistance, such as blockers of multidrug eﬄux pumps; and (3) using alternative antimicrobials such as bacteriophages. In addition, the strategy of finding new uses for existing drugs is beginning to produce results: antibacterial properties have been discovered for existing anticancer, antifungal, anthelmintic, and anti-inflammatory drugs. In this review, we discuss the antimicrobial properties of gallium compounds, 5-fluorouracil, ciclopirox, diflunisal, and some other FDA-approved drugs and argue that their repurposing for the treatment of bacterial infections, including those that are multidrug resistant, is a feasible strategy.

## Introduction

### New Approaches to Combat Bacterial Infections on the Verge of the Post-Antibiotic Era

Since the pharmaceutical industry has not produced a new class of antibiotics for more 6 than a decade (Walsh and Wencewicz, 2014) and the continuing rise of antibiotic resistance is a major health concern, the World Health Organization warned that we may enter a “post-antibiotic era” within this century, and they propose that urgent actions be taken (Aryee and Price, 2014). In addition, a recent estimate by the British government and the Welcome Trust warned that by the year 2050 there will be 300 million premature deaths and up to £64 trillion lost to the global economy due to bacterial infections. Therefore, research is now being directed toward (1) new types of antimicrobials, such as peptides (Seo et al., 2012), (2) novel therapeutic agents like bacteriophages (Abedon et al., 2011; Chan et al., 2013), (3) inhibiting bacterial virulence by blocking quorum sensing (QS; Kaufmann et al., 2008; Antunes et al., 2010; Rasko and Sperandio, 2010), and (4) targeting systems that confer resistance against antibiotics, such as multidrug eﬄux pumps (Lomovskaya and Watkins, 2001; Kaatz, 2002). Each of these approaches has faced significant difficulties. Antimicrobial peptides are expensive, toxic, and subject to proteolysis (Seo et al., 2012); bacteriophages are attacked by the immune system, and bacteria soon develop resistance to them (Brussow, 2012); and resistance against canonical QS inhibitors is possible (Maeda et al., 2012; García-Contreras et al., 2013b,c; Kalia et al., 2014).

Another approach to dealing with bacterial infections is to repurpose existing drugs as antibiotics or virulence inhibitors. This approach has several advantages, including avoiding the high costs and long development times for producing new antimicrobials, and also being able to utilize compounds with known pharmacological properties, and as is discussed here (see also **Table 1**), although the approach to find alternative antimicrobials by evaluating drugs with other approved uses is relatively new, there are at least a tens of feasible candidates with promising *in vitro* and *in vivo* (at least for some of them) antibacterial properties, being the diversity of the active compounds remarkable and their molecular targets diverse, and in some cases unexploited by conventional antimicrobials, it can be anticipated that a more extensive search for ideal candidates will continue to identify several yet undiscovered promising drugs, in fact recently, the UK and US launched programs to study de-prioritized drugs to find new therapeutic uses for them (Mullard, 2012). In this work we discuss the antimicrobial effects of attractive anticancer, anti-fungal, and anti-inflammatory drugs and argue that at least some of them are ideal candidates for repurposing as antibacterial agents, while others deserve further *in vitro* and *in vivo* research to fully evaluate their antibacterial potential.

**Table 1:** New strategies to combat bacterial infections.

Strategy	Examples	Advantages	Disadvantages
Develop new antibiotics	Daptomycin Novel oxazolidinone antibacterial agents	Optimized for inhibiting bacterial growth	Only two new clasess of antibiotics had been developed in the past 20 years Not a priority for the pharmaceutical companies Scaffolds are scarce
Develop new types of antimicrobials	Antimicrobial peptides Bacteriophages	New targets, new action mechanisms	Antimicrobial peptides: expensive, toxic, and subject to proteolysis Bacteriophages: attacked by the immune system, prone to develop resistance
Develop new anti-virulence drugs	Quorum sensing inhibitors Inhibitors of bacterial secretion systems	Targeting virulence instead of growth potentially could decrease resistance	Quorum sensing inhibitors: resistance mechanisms had been found Not effective against some clinical strains
Target resistance mechanisms	Multidrug efflux pumps inhibitors	Will allow the re-utilization of already non-effective antibiotics	Bacteria usually have several simultaneous drug resistance mechanisms
Drug repurposing for antibacterial and anti-virulence drugs	Ga containing compounds 5-FU Ciclopirox Diflunisal Statins Pentetic acid	Avoiding the high costs and long development times of novel antimicrobials Known pharmacological properties, known side effects, doses, interactions with other drugs, etc New targets, new action mechanisms Ample repertoire, high versatility Effective against several recalcitrant pathogens, including MDR.	Not optimized for antibacterial or antivirulence effects Ga containing compounds: their broad spectrum activity could be counterproductive damaging healthy host tissues, particularly fast growing cells with high iron acquisition 5-FU: high toxicity

### Anticancer Drugs as Antimicrobials

Due the similarities between cancer cells and pathogenic bacteria it is expected that some anti-neoplastics would be also effective against bacteria. In fact, the anticancer drugs daunorubicin and doxorubicin were originally isolated in 1939 as antibiotics. Nevertheless, they were found to be too toxic to be used for infections. In 1960, however, their antitumor properties were discovered (Weiss, 1992), and they are now utilized for the treatment of cancer. By the same token, some drugs that were originally developed as anti-neoplastics are effective at attenuating growth of microbial pathogens, and some of them have shown promising results in animal models and clinical trials. In the following section, the antimicrobial properties of gallium and 5-fluorouracil are discussed.

### Gallium

Gallium, a group IIIA element, has no known essential biological function but displays diverse biological activities. Many of gallium’s activities derive from its chemical similarities to ferric iron (Fe^3+^): Ga^3+^ and Fe^3+^ have remarkably similar ionic radii, electronegativities, electron, and ligand affinities, and coordination geometries (Bernstein, 1998). This allows Ga^3+^ to follow many of the uptake and transport pathways for Fe^3+^. It is the differences between Ga^3+^ and Fe^3+^, however, that are responsible for gallium’s therapeutic effects and low toxicity. Unlike Fe^3+^, which is easily reduced to a divalent state, Ga^3^ cannot participate in redox reactions under physiologic conditions. Ga will therefore not function in enzymes requiring iron, and will not become incorporated in heme, avoiding toxicity from interference with oxygen transport and cytochrome-mediated reactions (Hedley et al., 1988). Clinically, citrated Ga(NO_3_)_3_ for intravenous injection (Ganite^®^) is approved for use for the treatment of hypercalcemia of malignancy.

### Gallium in the Treatment of Cancer

In 1951 it was shown that ^72^Ga could be used for the early detection and palliative treatment of bone malignancies. In the 1970s, with the introduction of the radioisotope ^67^Ga, diagnostic gallium scintigraphy became widespread. ^67^Ga-scans are particularly sensitive to lymphomas, sarcomas, bone tumors, and hepatocellular carcinoma. Many of the ^67^Ga-avid cancers appear to be those that are most susceptible to therapeutic Ga treatment (Bernstein, 2013). Currently, ^67^Ga scanning is used in the diagnosis and staging of lymphomas. Ga(NO_3_)_3_ has demonstrated clinical efficacy in multiple myeloma, bladder cancer, lymphoma, ovarian cancer, and hepatocellular carcinoma (Bernstein, 1998; Bernstein et al., 2011).

### Gallium Compounds have Potent Antimicrobial Effects

The recognition of gallium as an antimicrobial was made in 1931 when it was used to treat experimental syphilis in rabbits and trypanosomiasis in mice (Levaditi et al., 1931). Surprisingly, there was a gap of more than 40 years until Ga was again reported to have antimicrobial activity (Srivastava et al., 1973). The antibacterial activity of Ga compounds both *in vitro* and *in vivo* has now been demonstrated against several important bacterial pathogens, such as Mycobacterium tuberculosis, Pseudomonas aeruginosa (Kaneko et al., 2007), Acinetobacter baumannii (Antunes et al., 2012; de Leseleuc et al., 2012), and Staphylococcus aureus. For a detailed description of gallium’s antibacterial effects, see the recent reviews by (Bernstein, 2013; Bonchi et al., 2014; Minandri et al., 2014) and **Table 2**.

**Table 2:** Antibacterial activities of the reviewed drugs.

Drug	Therapeutic use	Antibacterial activity *in vitro*	Antibacterial activity *in vivo*	Reference
Gallium compounds	Gallium nitrate: treatment of hypercalcemia of malignancy	Growth inhibition of several recalcitrant bacterial pathogens (including clinical isolates) such as: Gram (-) *Pseudomonas aeruginosa* y *A. baumanii* and Gram (+) like *Mycobacterium tuberculosis*	Effective against syphilis *(Treponema cuniculi)* in rabbits and trypanosomiasis in mice, guinea pigs, and monkeys Acute and chronic mice infections of *P. aeruginosa A. baumannii* infections in caterpillar and mice	Levaditi et al. (1931), Olakanmi et al. (2000), Kaneko et al. (2007), DeLeon et al. (2009), Antunes et al. (2012), de Leseleuc et al. (2012)
5-Florouracil	Anticancer drug Treatment of actinic keratosis and Bowen's disease	Growth inhibition of Gram (+) and (-) bacterial species Inhibition of biofilm formation Repression of QS *in P. aeruginosa*	Successful in clinical trials in humans, as external coating of central venous catheters	Hussain et al. (1992), Ueda et al. (2009), Walz et al. (2010)
Ciclopirox	Antifungal	Bacteriostatic and bactericide activity, depending on concentration used, in laboratory and clinical isolates	Not yet tested	Carlson-Banning et al. (2013)
5-fluorocytosine	Antifungal	Inhibition of virulence factor production by *P. aeruginosa*	Suppresses *P. aeruginosa* pathogenicity in a mouse model of lung infection	Imperi et al. (2013a)
Azithromycin	Antibiotic	Inhibition of QS systems of *P. aeruginosa*	Clinical evidence and clinical trials demonstrate efficacy in the treatment of chronic pulmonary infections of *P. aeruginosa*	Imperi et al. (2014)
Niclosamide	Antihelmintic	Inhibition of QS systems of *P. aeruginosa*	Reduction of *P. aeruginosa* pathogenicity in an insect model	Imperi et al. (2013b)
Diflunisal	Non-steroidal anti-inflammatory	Potent virulence inhibitor for the USA300 strain of methicillin-resistant *Staphylococcus aureus* (MRSA)	Not yet tested	Khodaverdian et al. (2013)
Statins	Lower plasma cholesterol levels	Reduction of virulence factor production of *P. aeruginosa* Broad spectrum antibacterial effects	Inhibition of *S. pneumoniae* attachment to lung and vascular tissue	Rosch et al. (2010)
Pentetic acid	Preparation of radiopharmaceuticals treatment of iron-storage disease and poisoning from heavy metals	Reduction of virulence factor production of *P. aeruginosa*	Effective to alleviate mice airway infections	Gi et al. (2014)
Terfenadine	Antihistamine	Inhibit growth of several pathogenic bacteria, including *S. aureus* and *M. tuberculosis*	Not yet tested	Perlmutter et al. (2014)
Zafirlukast	Treatment of asthma	Antimycobacterial	Not yet tested	Pinault et al. (2013)

### Mechanisms of Antimicrobial Activity for Gallium

Gallium is a non-redox Fe (III) analog that acts as a “Trojan horse” due to its chemical similarity to Fe(III). In Menon et al. (1978) reported that Ga is taken up by several bacteria and fungi. Later it was shown that *Pseudomonas fluorescens* internalized Ga (Al-Aoukaty et al., 1992) and it was suggested that the transport of Ga into *P. aeruginosa* does not involve its main iron transporters, the siderophores pyoverdine and pyochelin (Kaneko et al., 2007); although recently it was discovered that the pyochelin system is likely able to transport moderate gallium concentrations (Frangipani et al., 2014). In addition, Ga inhibits Fe(III) transport through interference with pyoverdine: by decreasing its production, through interference with the *pvdS* sigma factor, and by directly binding pyoverdine and hence decreasing Fe(III) uptake (Kaneko et al., 2007).

Currently, other than pyochelin, HitAB is the only Ga transporter known in *P. aeruginosa* (García-Contreras et al., 2013a). However, *hitA/hitB* mutants of *P. aeruginosa* still can take up Ga, suggesting the existence of secondary Ga transporters (García-Contreras et al., 2013a), Although pyochelin delivers iron trough the outer membrane transporter, FptA, the possibility that it also internalizes it via the HitB membrane protein remains to be explored. Due to its similarity to iron, it is hypothesized that Ga inhibits several Fe-redox dependent processes, such as iron transport, respiration, DNA replication, and reactive oxygen species (ROS) protection. Inhibition of Fe(III) transport by Ga was first demonstrated for intraphagosomal *M. tuberculosis* (Olakanmi et al., 2000). The same group demonstrated that for *Francisella novicida* and *Francisella tularensis*, Ga inhibits iron acquisition from lactoferrin or transferrin *in vitro* and *in vivo* in macrophages (Olakanmi et al., 2010). Regarding the effects of Ga on protection against ROS, it was shown that for *F. tularensis,* the addition of 10 μM Ga-transferrin or Ga-lactoferrin decreased up to 70% the activity of Fe-containing catalase and Fe-cofactored SOD, rendering the cells more sensitive to H_2_O_2_. In *P. fluorescens*, prolonged exposure to Ga decreased the iron content in catalase and its activity (Beriault et al., 2007). Also, for *P. fluorescens,* Ga disrupted oxidative phosphorylation by inhibiting the respiratory complexes. In addition, Ga inactivated aconitase and fumarase A, two Fe-dependent enzymes (Chenier et al., 2008). The effects of Ga on the global metabolism of *P. aeruginosa* were recently investigated by metabolomics: 64 metabolites showed significant changes between Ga-free and Ga-citrate cells (Rzhepishevska et al., 2011).

Due to the very promising antibacterial effects of Ga, the repurposing of Ga(NO_3_)_3_ and other Ga compounds for treating MDR bacterial infections has been suggested recently (Bonchi et al., 2014). In addition, considerable progress has been recently made in the development of Ga delivery technologies, developing materials like gallium-doped phosphate-based glasses (Valappil et al., 2009) and gallium-carboxymethyl cellulose (Valappil et al., 2013). These materials enable controlled Ga release and are effective at killing *P. aeruginosa* and, in the case of the glasses, other important pathogens such as MRSA and *Clostridium difficile*. Other promising biomaterials that incorporate gallium are mesoporous bioactive glass scaffolds (Shruti et al., 2013), Ga-containing phosphosilicate glasses (Franchini et al., 2012; Lusvardi et al., 2013) and Ga grafted titanium implants which decrease biofilm formation of oral bacteria such as *Streptococcus mutans* (Cochis et al., 2015). Such novel materials may eventually be used in other clinical applications, for example to coat catheters and medical prosthetic devices.

### Bacterial Adaptation and Resistance to Gallium

One of the main mechanisms of gallium’s antibacterial activity may be the generation of ROS (Beriault et al., 2007; Chitambar, 2010). Consistent with this effect, *P. fluorescens* exposed to 1 mM Ga-citrate has high amounts of oxidized lipids and proteins. Such cells, however, respond to Ga by increasing the synthesis of enzymes that produce NADPH and by increasing SOD (Beriault et al., 2007). An increase in NADPH would provide the reductive power for the detoxification of ROS by glutathione peroxidase/reductase (Perry et al., 1991) and would protect catalase from inactivation by H_2_O_2_ (Kirkman et al., 1999). In addition, in the presence of Ga, *P. fluorescens* compensates for the loss of its fumarase A activity by overexpressing Fe-independent fumarase C. In order to maintain the oxidation of NADH, an NADH oxidase that is absent in the control cells is induced (Chenier et al., 2008). Also, pyocyanin overexpression helps cells cope with Ga toxicity (García-Contreras et al., 2013a). This protection may be due its capacity to reduce Fe(III) to Fe(II), which would not be affected by Ga competition. Accordingly, lower concentrations of Fe(II) than of Fe(III) are needed to protect the cells against Ga (García-Contreras et al., 2013d). This fact may be clinically relevant, since Fe(II) is increasingly available to *P. aeruginosa* during the course of an infection, and it promotes pulmonary infections in CF patients (Hunter et al., 2013).

Moreover, the overexpression of the siderophore pyoverdine in *P. aeruginosa* may also promote gallium resistance, since it is likely able to sequester extracellular Ga, preventing its entrance to the cell. Adding pyoverdine to cultures increases their Ga tolerance (Kaneko et al., 2007; Frangipani et al., 2014). In contrast, it was recently demonstrated that the disruption of pyochelin biosynthesis and uptake increases the MIC50 for gallium two–fourfold in the *P. aeruginosa* PAO1 strain (Frangipani et al., 2014). Interestingly, restricting gallium’s growth inhibitory effects by pyoverdine sequestration strongly decreases the development of bacterial resistance (Ross-Gillespie et al., 2014).

Another important property of Ga compounds is that they modulate the expression of several virulence factors in *P. aeruginosa*. For example, they decrease the expression of pyoverdine (Kaneko et al., 2007; García-Contreras et al., 2013d). Depending in the growth conditions, concentrations, and perhaps on the particular gallium compound, gallium can either decrease (Kaneko et al., 2007; Valappil et al., 2013) or promote (García-Contreras et al., 2013d) biofilm formation. Although the molecular processes involved in the modulation of virulence factors by gallium are still unknown, gallium’s role may be related to its ability to create a state of iron deficiency, since iron starvation strongly promotes virulence (García-Contreras et al., 2013d). Although Ga has proven effective to alleviate diverse animal infections, whether growth sub-inhibitory concentrations can promote bacterial virulence *in vivo* remains unexplored.

There is thus substantial evidence demonstrating that gallium compounds have a broad spectrum of antibacterial activity, and their mechanisms of action are fairly well defined. Clinical trials to test Ga(NO_3_)_3_ in patients with cystic fibrosis are being conducted at the University of Washington; these trials are being designed to assess the pulmonary safety of the compound and its efficacy in improving lung function and decreasing *P. aeruginosa* counts.

### 5-Fluorouracil

The uracil analog 5-fluorouracil (5-FU) is an antimetabolite developed in by Heidelberger et al. (1957). Twenty years later, clinical trials confirmed that 5-FU was an effective anticancer drug. To date, 5-FU is widely used in the clinic, being applied systemically to treat gastrointestinal adenocarcinoma and squamous cell carcinoma, or as adjuvant chemotherapy for breast carcinoma and others. It is also applied topically to treat actinic keratosis, superficial basal cell carcinoma, and Bowen’s disease.

### 5-Fluorouracil as an Antibacterial, Antibiofilm, and Virulence Inhibitor

In 1985, it was shown that 5-FU had potent antimicrobial effects against several bacterial pathogens. It was then confirmed that 5-FU inhibited the growth of *S. aureus* and *S. epidermidis*, with an MIC50 ≤0.8 μg/mL, and that it acts synergistically against Gram (-) bacteria when combined with beta-lactamics (Gieringer et al., 1986). Later, it was found that it is synergistic with tobramycin against *S. aureus* (Nyhlen et al., 2002) and that it inhibits *S. epidermidis* biofilms (Hussain et al., 1992).

Unlike conventional antimicrobials, which act by directly affecting bacterial growth and survival, anti-virulence therapies are designed to decrease the potential damage produced by the pathogens to the host, hence reducing the chances for the development of resistance (Rasko and Sperandio, 2010). In this regard, it was found that because uracil biosynthesis is important for QS, swarming, biofilm formation, and production of QS-controlled virulence factors (Ueda et al., 2009), 5-FU is an effective repressor of QS and biofilms without having a severe inhibitory effect on growth, and was able to protect barley against *P. aeruginosa* infection. The reduction of *P. aeruginosa* pathogenicity by 5-FU was also later reported by Imperi et al. (2013a) when they demonstrated that 5-fluorocytosine, which is converted to the active compound 5-FU, also reduces pyoverdine, PrpL protease, and exotoxin A, and suppresses *P. aeruginosa* pathogenicity in murine lung infections. 5-FU is also able to inhibit biofilms and virulence of enterohemorrhagic *Escherichia coli* (Attila et al., 2009). Remarkably, in Walz et al. (2010) 5-FU was used in large-scale human trials as an anti-infective external coating of central venous catheters, producing better results than the positive controls chlorhexidine and silver sulfadiazine. It was thus demonstrated that 5-FU provides a safe and effective coating on catheters for critically ill patients (Walz et al., 2010).

The effect in 5-FU during bacterial infections *in vivo* probably would be dual, targeting both the production of virulence factors and biofilms as well as bacterial growth. Toxicity to bacteria likely will depend on the available nutrients, particularly uracil, since *in vitro* in rich culture medium (with 0.2 mM of uracil), *P. aeruginosa* can grow well with 25–50 μM of 5-FU (Ueda et al., 2009; García-Contreras et al., 2013c), while similar concentrations of 5-FU are able to prevent its growth in minimal medium with no added uracil (West and Chu, 1986).

### 5-Fluorouracil Action Mechanisms and Resistance in Bacteria

Although the effects of 5-FU as an anticancer drug are well known, its effects as an antibacterial have not been extensively studied. In 1961, one study showed that 5-FU inhibits ribosome and protein synthesis in Mg-deprived bacteria. In fungi, 5-FU is transformed to 5-fluorouridine triphosphate (FUTP) and then incorporated into RNA instead of uridylic acid, altering the amino-acylation of tRNA, disturbing the amino acid pool and inhibiting protein synthesis (Waldorf and Polak, 1983; Vermes et al., 2000). In addition, 5-FU is also converted into 5-fluorodeoxyuridine monophosphate (FdUMP), a potent inhibitor of thymidylate synthetase, an enzyme essential for the generation of thymidine, and its inhibition prevents DNA synthesis (Vermes et al., 2000).

Although the mechanisms of action for 5-FU against QS and biofilms are largely unknown, inhibition of *E. coli* biofilm formation by 5-FU requires the presence of AriR (Attila et al., 2009), which binds DNA and supresses biofilms (Lee et al., 2007). Not surprisingly, along with the lack of mechanistic studies about 5-FU’s effects on bacteria, studies of adaptation and resistance to it are scarce. Recently, however, it was shown that although 5-FU inhibits the QS-controlled virulence factor production of *P. aeruginosa*, some clinical strains are resistant (García-Contreras et al., 2013c).

Similarly to gallium compounds, 5-FU has broad spectrum antimicrobial activity and inhibits virulence factor and biofilm production. Since it had been shown effective at preventing microbial colonization of catheters in human trials, we expect that it could be eventually implemented for this purpose, and that other possible uses, such as a topical or systemic antibiotic, could be tested in animal models soon.

### Repurposing Antibiotics as Virulence Inhibitors

Interestingly, several different antibiotics, particularly macrolides such as azithromycin but also ceftazidime (CFT) and ciprofloxacin, are able to decrease the expression of QS controlled virulence factors of *P. aeruginosa,* and azithromycin has improved the clinical outcome of chronically infected cystic fibrosis patients (Skindersoe et al., 2008). In addition, this antibiotic has anti-inflammatory properties and is considered an ideal candidate for repurposing as an anti-virulence agent. For a detailed description of azithromycin as an anti-virulence drug, see (Imperi et al., 2014). It is also noted that ciprofloxacin, metronidazole, and tinidazole may interfere with QS *in vivo* when used to treat Crohn’s disease (Struss et al., 2012).

### Antifungals and Other Types of Drugs

Some antifungal drugs also have potent antibacterial activity. Deng and collaborators identified a compound with broad-spectrum antibacterial activity, which later led to the identification of ciclopirox as an antibacterial, by a structural based search (Carlson-Banning et al., 2013). Ciclopirox is an off-patent, topical antifungal developed 40 years ago. It is notable for its excellent safety profile; also, unlike many antifungals, no fungal resistance to it has been identified (Subissi et al., 2010), which is remarkable. The antifungal mechanism of ciclopirox remains unknown, though it is known to not inhibit ergosterol biosynthesis (Niewerth et al., 2003), and likely interferes with iron metabolism (Sigle et al., 2005). Ciclopirox has potent bacteriostatic activity against laboratory and clinical isolates of *E. coli*, including ciprofloxacin resistant isolates, with an MIC50 of 5–15 μg/mL; at higher concentrations, it acts as bactericide. Moreover, it also inhibits growth of *A. baumannii, Klebsiella pneumoniae, P. aeruginosa* (including MDR isolates), and *Proteus mirabilis.* The activity of ciclopirox against *E. coli* involves interference with galactose metabolism and disruption of LPS biosynthesis. For *P. aeruginosa*, ciclopirox inhibits pyocyanin and may increase pyoverdine production (Carlson-Banning et al., 2013).

The anthelmintic drug niclosamide inhibits *P. aeruginosa*’s production of acyl-homoserine lactone, QS signaling molecules, and QS-regulated virulence factors. Niclosamide reduced biofilm formation and motility, preventing *P. aeruginosa* pathogenicity in insects (Imperi et al., 2013b). Similarly, the non-steroidal anti-inflammatory drug diflunisal is a potent virulence inhibitor for the USA300 strain of methicillin-resistant *S. aureus*. It inhibits the production of alpha-hemolysin and modulin α without inhibiting growth (Khodaverdian et al., 2013). In addition, statins, which lower plasma cholesterol levels, also reduce virulence factors of *P. aeruginosa* when used at 10–100 μM (Hennessy et al., 2012), *in vivo* attachment of *S. pneumoniae* to lung and vascular tissue at 1 μM (Rosch et al., 2010) and have broad spectrum antibacterial effects at relatively high concentrations 15–600 μM (Hennessy et al., 2012), making them additional candidates for repurposing as antibacterial agents. Nevertheless, the antibacterial properties of these drugs have still not been demonstrated in mice or other mammals, and no clinical trials have been conducted.

Other interesting compounds with potential to be repurposed as anti-virulence drugs are: (1) pentetic acid, a chelating agent used in the preparation of radiopharmaceuticals and to treat iron-storage disease and poisoning from heavy metals, which is able to decrease elastase activity, PQS production, and biofilm formation of *P. aeruginosa* (Gi et al., 2014); (2) the antihistamine terfenadine, which inhibits bacterial type II topoisomerases and thus has potent antimicrobial activity against *S. aureus*, *M. tuberculosis* and other bacterial pathogens (Perlmutter et al., 2014); and (3) zafirlukast, a leukotriene receptor antagonist used in the prophylactic treatment of asthma, which inhibits growth of *M. tuberculosis* by blocking complex formation between the protein Lsr2 and DNA, hence interfering with the condensation and structural organization of the bacterial genome; interestingly, this drug target is novel since no current anti-mycobacterial agent is directed against it (Pinault et al., 2013).

## Concluding Remarks

Based on the evidence discussed (summarized in **Table 2**), there are currently several drugs in clinical use for other indications that have demonstrated *in vitro* efficacy at inhibiting the growth and/or virulence of bacterial pathogens. These drugs are structurally very diverse and have a very broad spectrum of clinical uses. Most of these drugs show activity against clinical isolates, including those that are multidrug resistant and already practically untreatable, such as MDR isolates of *P. aeruginosa* (Poole, 2011), A. *baumannii,* and other recalcitrant bacterial pathogens (Livermore, 2009); moreover, some of them have alleviated infections in animal models and the possible ways bacteria may adapt and develop resistance against some of the most promising drug candidates is at least partially known. 5-fluorouracil has shown remarkable antibacterial efficacy in clinical trials, while the other very promising drug candidates, including gallium compounds, are either still awaiting or are just beginning human clinical antibacterial testing. We propose that these compounds represent new opportunities in the battle against increasingly antibiotic-resistant bacteria, and we encourage the research and medical communities to further advance their implementation as antibacterial drugs in the clinic. We also encourage the evaluation of as-yet untested drugs as potential antimicrobial and antivirulence compounds: we feel this is a fertile field to find new antimicrobials that could be brought quickly into clinical use. Although some potential difficulties and disadvantages of repurposing drugs exist (see **Table 1**), drug repurposing may be the only feasible alternative to effectively treat bacterial infections in a relatively short period of time, before we have to return to a pre-antibiotic era.

## Conflict of Interest Statement

The authors declare that the research was conducted in the absence of any commercial or financial relationships that could be construed as a potential conflict of interest.
